# Structure based comprehensive modelling, spatial fingerprints mapping and ADME screening of curcumin analogues as novel ALR2 inhibitors

**DOI:** 10.1371/journal.pone.0175318

**Published:** 2017-04-11

**Authors:** Sant Kumar Verma, Suresh Thareja

**Affiliations:** School of Pharmaceutical Sciences, Guru Ghasidas Central University, Bilaspur, C.G., India; Istituto di Genetica Molecolare, ITALY

## Abstract

Aldose reductase (ALR2) inhibition is the most legitimate approach for the management of diabetic complications. The limited triumph in the drug development against ALR2 is mainly because of its close structural similarity with the other members of aldo-keto reductase (AKR) superfamily *viz*. ALR1, AKR1B10; and lipophilicity problem *i*.*e*. poor diffusion of synthetic aldose reductase inhibitors (ARIs) to target tissues. The literature evidenced that naturally occurring curcumin demonstrates relatively specific and non-competitive inhibition towards human recombinant ALR2 over ALR1 and AKR1B10; however β-diketone moiety of curcumin is a specific substrate for liver AKRs and accountable for it’s rapid *in vivo* metabolism. In the present study, structure based comprehensive modelling studies were used to map the pharmacophoric features/spatial fingerprints of curcumin analogues responsible for their ALR2 specificity along with potency on a data set of synthetic curcumin analogues and naturally occurring curcuminoids. The data set molecules were also screened for drug-likeness or ADME parameters, and the screening data strongly support that curcumin analogues could be proposed as a good drug candidate for the development of ALR2 inhibitors with improved pharmacokinetic profile compared to curcuminoids due to the absence of β-diketone moiety in their structural framework.

## Introduction

Diabetes mellitus (DM), a common metabolic disorder designated by the hyperglycaemic state, adversely affects the homeostasis of various organ systems [1]. Long-term hyperglycaemia causes acute reversible and chronic cumulative irreversible changes, includes damage to blood vessels and peripheral nerves which eventually leads to diabetic complications such as vasculopathy, nephropathy, neuropathy, retinopathy, and cataracts; greatly increasing the risk of atherosclerosis, heart attack, stroke, blindness, amputation, and kidney failure [2, 3]. Worldwide, 387 million peoples are living with diabetes with the prevalence of 8.3% *i*.*e*. one person in twelve is suffering from diabetes. In the year 2014, 4.9 million individuals died from diabetes with the death rate of one person per seven seconds. The diabetes expenditure has reached 612 billion US$ in the same year [4]. If the current demographic pattern continues, the diabetic populations will increase more than 592 million up to the year 2035 [5]. The direct economic cost of diabetes is about 10% of the total health care budget of National Health Service (NHS) and is projected to account for around 17% in 2035/2036; furthermore, approximately 90% of the total direct cost is needed for the treatment of the devastating diabetic complications [6].

**Fig 1:**
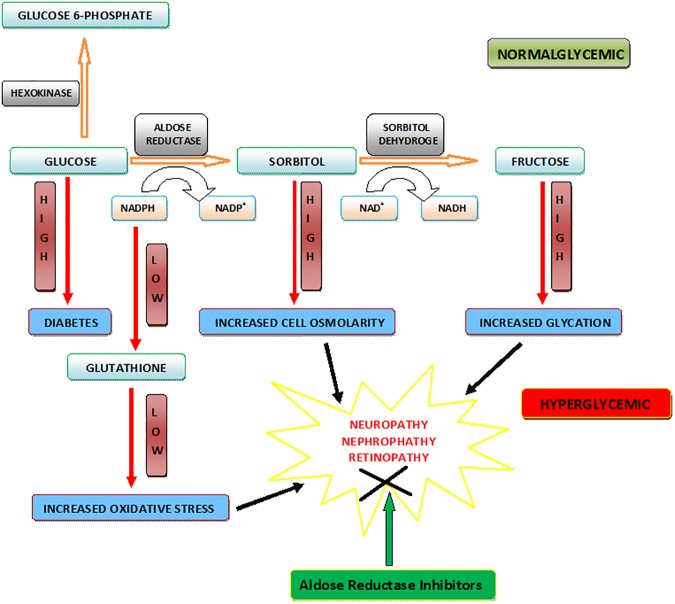
ALR2 mediated Polyol pathway.

Although tight control of blood glucose reduces the incidence of diabetic complications, a significant fraction of diabetic patients with good glycaemic control still shows the devastating complications associated with diabetes [7]. Despite advances in the treatment of diabetes, it is still difficult to prevent the development and progression of many of the disabling complications associated with this disease [2]. Several mechanisms for the pathogenesis of diabetic complications have been proposed such as the polyol pathway [8], non-enzymatic glycation [9], protein kinase C (PKC) [10], hexosamine [11], and overproduction of superoxide by the mitochondrial electron transport chain [12]. Evidences have demonstrated a link between enhanced metabolism of glucose through the polyol pathway (Fig 1) and the onset and progression of long-term diabetic complications [7].

In normal glycaemic condition, glucose enters into glycolysis cycle; leading to the production of pyruvate and energy. In hyperglycaemic condition, the excess of glucose enters into polyol pathway *via* aldose reductase (AR, ALR2) enzyme. Aldose reductase, a key member of the aldo-keto reductase (AKR) superfamily, is the first and rate-limiting enzyme of the polyol pathway, a glucose-shunt that channels excess glucose to form fructose through sorbitol in hyperglycaemic condition [13]. In polyol pathway, AR initially catalyses the stereospecific transfer of a hydride from NADPH to the aldehyde form of glucose to form sorbitol. Sorbitol dehydrogenase, in turn, utilizes NAD^+^ and oxidizes this intermediate polyol to fructose (Fig 1).

Diabetic complications arise mainly due to prolonged exposure of the body to high concentrations of glucose. During hyperglycaemia, there is an increased flux of glucose in polyol pathway. More than 30% of the glucose is metabolized by polyol pathway during diabetes conditions (less than 3% in normoglycaemic conditions) [14]. Under hyperglycaemia, increased polyol pathway activation leads to the production of excess sorbitol which is impermeable through biological membranes, accumulates inside the cells, and causes osmotic stress leading to secondary diabetic complications [15]. Further, in hyperglycaemia, increased utilization of NADPH (reduced form of nicotinamide adenine dinucleotide phosphate) by AR could result in decreased supply of NADPH co-factor to glutathione reductase that converts glutathione disulfide (GSSG) to glutathione (GSH) leading to decreased GSH reductase activity and in turn decreased GSH levels (Fig 1). Since it is well known that decreased GSH levels contribute to oxidative stress, AR-mediated increases in NADPH consumption could also lead to oxidative stress [16]. The hyperglycaemic injury is in part due to osmotic and oxidative stress, induced by AR-mediated reduction of glucose to sorbitol. Further, support for a critical role of AR in mediating the toxic effects of glucose is provided by the demonstration that overexpression of AR in the lens of transgenic mice accelerates diabetic cataracts [17]. It has also been demonstrated that high glucose in diabetes leads to the up-regulation of AR in several tissues and the treatment with specific AR inhibitors prevents hyperglycaemia-induced hyperplasia and hyper proliferation of vascular smooth muscle cells [18]. Hyperglycaemia causes proliferation of vascular smooth muscle cells and apoptosis of vascular endothelial cells. These observations indicate that AR inhibition could be useful in preventing the pro-vascular-proliferative effects of diabetes, which is still remain the major cause of morbidity and mortality associated with this disease.

**Fig 2:**
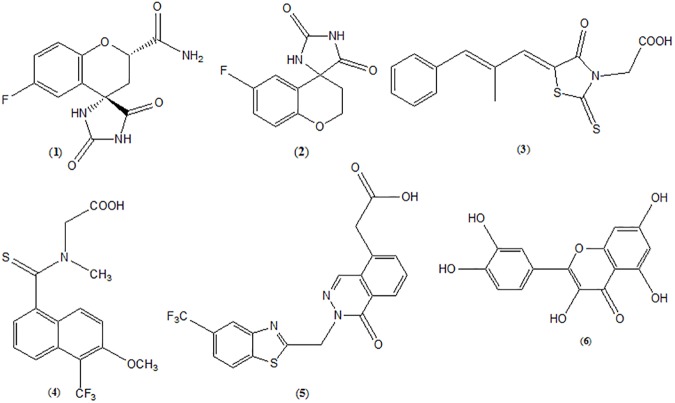
ARIs of synthetic (1–5) and natural origin (6) developed during last few decades.

*In vivo* animal studies performed by different researchers using synthetic and natural compounds as AR inhibitors favour that AR inhibition could be effective for management of diabetic complications, and some of them have been evaluated in clinical trials [19, 20]. During the last decade, numbers of aldose reductase inhibitor (ARI) have been developed (Fig 2) which mainly include hydantoins, e.g. Fidarestat (1) and Sorbinil (2); carboxylic acid derivatives, e.g. Epalrestat (3), Tolrestat (4) and Zopolrestat (5); and molecule of natural origin, e.g. Quercetin (6).

To date, Epalrestat is the single drug molecule available in the market for the treatment of diabetic peripheral neuropathy [21, 22]. Fidarestat and Ranirestat are other molecules which have reached to advanced phase of clinical trials [23, 24]. Further, most ARIs developed so far have acquired limited triumph, among them in particular synthetic ARIs were facing lipophilicity problem *i*.*e*. poor diffusion to target tissues such as retina and nerve, along with linked harmful side effects [20, 25].

Natural products containing inherently vast structural diversity than synthetic compounds are the major sources of bioactive agents, and will continually play leading role in discovering new drugs. Phytochemicals are considered privileged structures as they have the diversity space in which chemical scaffolds embody characteristics that promote binding to multiple protein targets. An analysis of the origin of the drugs that were launched in the last 25 years showed that both natural products and their derived semi-synthetic compounds composed 34% of all new chemical entities, while 18% of them were synthetic mimics of natural compounds [26, 27].

**Fig 3:**
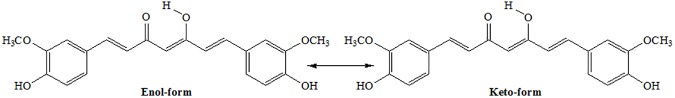
Tautomeric forms of curcumin.

Curcumin, a natural polyphenolic diarylheptanoid obtained from the dried rhizome of the herb *Curcuma longa* Linn. It is evident from the literature that curcumin is a multi-target pleiotropic agent, showing a broad range of biological activities. Turmeric (*Curcuma longa*) has been widely used in India and China as a spice, dietary pigment and in traditional medicine, such as remedies against the diabetic complications [28–30]. Naturally occurring curcumin demonstrates relatively specific and non-competitive inhibition towards human recombinant ALR2 over another structurally similar members of AKR superfamily *i*.*e*. aldehyde reductase (ALR1) and human small intestine reductase (HSIR, AKR1B10) with IC_50_ value 6.8 μM [31], which is nearly 5-fold lesser than that of quercetin (IC_50_ = 37.6 μM) [32], a well-known ARI of natural origin. More importantly, curcumin also prevents the accumulation of intracellular sorbitol under hyperglycaemic state, in turn, diminishes the osmotic cellular stress, resulting into delay in progression of diabetic complications [31, 33, 34]. Two cumulative α, β-unsaturated carbonyl groups work as a linker between both aromatic phenol rings present in curcumin, and both of them exhibit keto-enol tautomerization *via* an enolate intermediate (Fig 3). Under the neutral pH conditions, curcumin predominantly exists as a keto form [31]; however β-diketone moiety of curcumin is a specific substrate for liver aldo-keto reductases and may be accountable for it’s rapid *in vivo* metabolism [35]. Various structural modifications were made by different researchers in the hemical structure of curcumin to improve its pharmacokinetic profile [36–39].

Taking into consideration, the ALR2 selectivity and inhibitory potential of curcumin, an integrated molecular docking assisted three-dimensional quantitative structure activity relationship (3D-QSAR) models were developed on a data set of 21 molecules comprises of naturally occurring curcuminoids and synthetic curcumin analogues active against ALR2. Molecular docking (MD) typically uses an energy-based scoring function to identify the energetically most favourable ligand conformation when bound to the target. It predicts the binding affinity and explores the binding mode of interactions of ligands with the key amino acid residues present at the active binding site of the target [5]. 3D-QSAR models are essential for the generation of a pharmacophore required to facilitate molecular recognition and binding. The primary aim of a 3D-QSAR technique is to establish a correlation of biological activities of a series of structurally and biologically characterized compounds with the spatial fingerprints of the major field properties of each molecule, such as steric and electrostatic potential [5, 40]. Further, a three-dimensional pharmacophore was generated as an outcome of 3D-QSAR studies, the generated spatial fingerprints or pharmacophoric features mapped and can be used for the designing and development of newer ALR2 selective curcumin analogues with high potency as well as improved pharmacokinetic profile for the management of diabetic complications.

## Methodology

### Data set and biological activity

**Fig 4:**
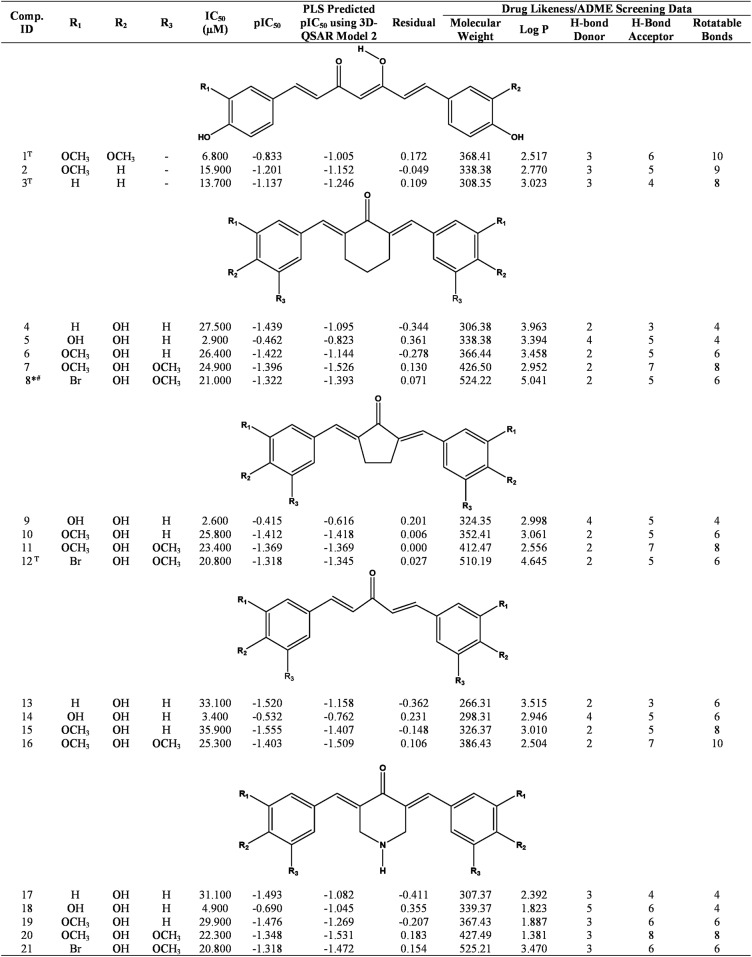
Chemical structure of curcuminoids (compound 1–3), synthetic curcumin analogues (compound 4–21) along with their observed ALR2 inhibitory activity (IC_50_), pIC_50_, predicted pIC_50_, residual activity, and drug likeness/ADME screening Data. ^T^Test set compounds; molecule violating drug-likeness/ADME screening due to: *molecular weight > 500, and ^#^Log P > 5.

A dataset of 21 molecules comprises of curcuminoids obtained from *Curcuma longa* (compound 1–3), and synthetic curcumin analogues (4–21) was selected for present study [32]. Data set was split into training set of 18 molecules, and test set of 3 molecules. The division was done in such a manner that three compounds (1, 3 and 12) shuffled in the test set, representing the structurally divergent features of molecules present in training set with a wide range of ALR2 inhibitory potential. However, the final compounds in training and test sets were decided based on the highest Q^2^ and R^2^ values with 85% compounds in training set and the remaining compounds in test set. The reported IC_50_ values of data set molecules were converted into pIC_50_ (pIC_50_ = −log IC_50_) to arrange the data in ascending linear manner for the QSAR analysis (Fig 4).

### Molecular modelling, docking, and alignment

The present molecular modelling studies were accomplished with the use of different software packages namely Molegro Virtual Docker (MVD 6.0.0 2013) [41], VLife MDS 3.5 [42], SOMFA 2.0.0 [43], TSAR 3D 3.3 [44], and Vega ZZ 3.0.3.18 [45]. Firstly, the structures of data set molecules were drawn using ChemDraw Ultra 8.0; then these sketched molecules were converted into 3D and subjected to energy minimization to attain the stable conformation with the lowest energy using Chem3D Ultra 8.0. The geometrical optimization was performed with the subsequent use of dual optimizers *viz*. molecular mechanics (MM2) followed by Hamiltonian approximation (AM1) available in MOPAC module. The implicit solvent environment or solvent effect was taken for geometrical optimizations which replace the explicitly represented water molecules with a mathematical expression that reproduces the average behaviour of water molecules [5]. The geometrical optimization process was run till the root-mean-square (RMS) gradient value reaches a value lesser than 0.001 kcal/mol Å in both the optimization techniques mentioned above [1].

**Fig 5:**
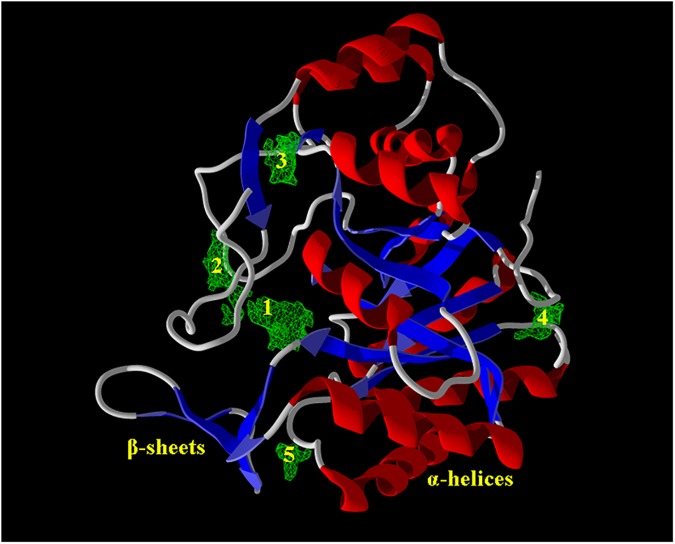
Predicted binding cavities (1–5) (green) within ALR2 (secondary structure).

The geometrically optimized conformers were imported into the workspace of MVD (MVD 2013.6.0 evaluation version) along with the ALR2 (PDB entry: 4JIR, ALR2 from *Homo sapiens* and co-crystalized with Epalrestat as well as NADP^+^) [46]. While retrieving target molecule from protein data bank (PDB), the associated water molecules were eliminated, and NADP^+^ imported as co-factor. All the molecules in the workspace were subjected to molecular preparation to assign missing bonds, bond orders, hybridization, charge, explicit hydrogens, tripos atom types and detect flexible torsions in ligands. Potential binding sites also referred to as cavities or active sites (1–5) (Fig 5) were identified using the built-in cavity detection algorithm. During this computational process, the maximum numbers of cavities were fixed to 5, grid resolution 0.80 Å, minimum cavity volume 10 Å^3^, maximum cavity volume 10,000 Å^3^ and probe size 1.2 Å; while the other parameters were kept as default [1].

**Fig 6:**
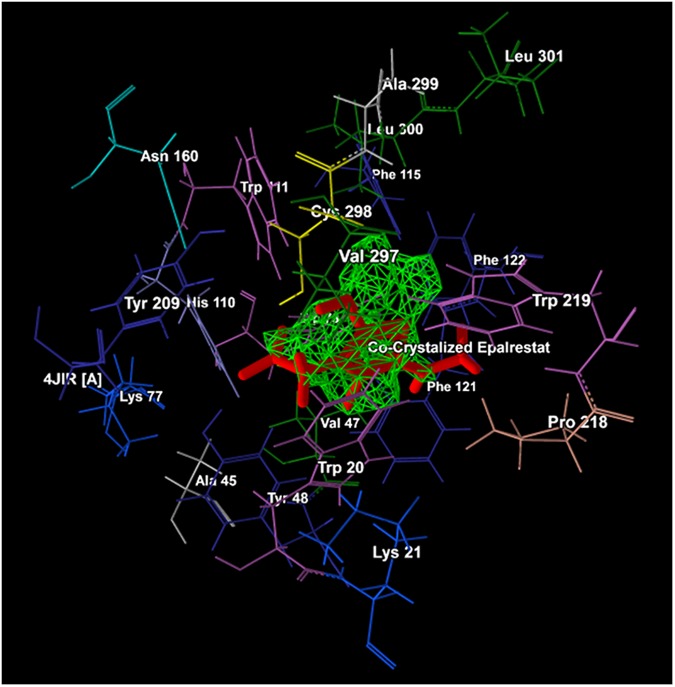
Cavity 1 (green) with co-crystalized epalrestat (red) and its corresponding amino acid residues.

MD simulations were commenced into the largest cavity (1) corresponding to co-crystalized Epalrestat binding cavity of 4JIR (Fig 6) to determine the binding affinity and binding mode of interactions of data set molecules with ALR2. A synthetic ARI presently available in the market *viz*. Epalrestat was taken as a reference to check the accuracy and reliability of MD simulations, and the binding affinities of data set molecules were also compared with an ARI of natural origin namely Quercetin in terms of docking scores. For the evaluation of docking solutions, grid based scoring function MolDock Score [47] was selected at 0.3 Å grid resolutions. MolDock Simplex Evolution (MolDock SE) search algorithm with number of runs 10 and population size 50 was selected for performing MD simulations [1]. The number of runs specifies the number of times that the docking simulation was repeated for each ligand chosen to be docked and each of these runs was returning to a single final solution *i*.*e*. pose. The only negative lowest-energy representative cluster was returned from each of them after completion of docking, and the similar poses were removed keeping the best scoring one. The clusters were ranked through the simple comparison between the conformations of the lowest binding energy in each cluster. The other parameters such as maximum iterations, energy threshold, binding radius, SE maximum steps and SE neighbouring distance factor were set to 1,500, 100, 15 Å, 300 and 1.00, respectively. For cluster similar poses as well as ignore similar poses (for multiple runs only), the RMSD threshold was fixed 1.00 Å [1]. The pose or conformation of each ligand with the highest MolDock score was selected for the analysis of its’ steric and hydrogen bond interactions with ALR2.

Further, the lowest binding energy conformers of all the data set molecules (1–21) obtained from MD simulations were aligned separately by two different alignment approaches based on molecular weighted (MW) extent and moments of inertia (MI) using TSAR package. The MW and MI aligned structures were exported into.cssr file format and further converted to.cTF2 using file format converter present in SOMFA package because it is readily taken by SOMFA software.

### Generation of 3D-QSAR models

The 3D-QSAR models were constructed on aligned molecules obtained from MW (model 1, 2) and MI (model 3, 4) alignment approaches. The external applicability domain *i*.*e*. test set of 3D-QSAR models was built by compounds 1, 3 and 12. All the molecules in.cTF2 file format were loaded along with their biological activity (pIC_50_) against ALR2 into the workspace of the 3D-QSAR package. Only training set molecules were used in the development of 3D-QSAR models. The 3D-QSAR models were generated with 40 x 40 x 40 Å grid originating at (-20, -20, -20) with resolution of 0.5 Å and 1.0 Å both [5, 40]. The steric and electrostatic properties of the data set molecules generated from 3D-QSAR software against ALR2 were used for the development of 3D-QSAR models. The three-dimensional steric and electrostatic master grid maps were generated from 3D-QSAR at definite grid resolutions; represent the area in space where steric and electrostatic field interactions are responsible for the observed biological activity. An individual compound in the data set can be visualized in these grids and variation in activity can be best explained by the grids [5]. Further, the generated models were used for prediction of the ALR2 inhibitory potential of all the data set molecules.

### Regression analysis by PLS method

Partial least square (PLS) in conjugation with leave one out (LOO) cross-validation techniques implemented in VLife MDS was used for the regression analysis of developed 3D-QSAR models, in which the steric and electrostatic properties of data set molecules were independent variable and pIC_50_ values were used as dependent variables. These properties were correlated with biological activity to identify the three-dimensional molecular properties responsible for selective and potent ALR2 inhibitory activity [5, 40].

### Validation of developed 3D-QSAR models

The statistical fitness of developed 3D-QSAR models was evaluated under various statistical parameters obtained from PLS regression analysis, such as cross-validated correlation coefficient (q^2^) as an internal statistical index of predictive power (Formula 1), correlation coefficient (r^2^) external predictivity indicator (Formula 2), predictive correlation coefficient r^2^_pred_ (Formula 3), standard error of estimate (S-value) (Formula 4) and Fischer statistics (F-test) (Formula 5) [40].

$$q^{2}=1\;\text{-}\;\frac{\sum {\left(Y_{cal}\;\text{-}\;Y_{obs}\right)}^{2}}{\sum {\left(Y_{obs}\;\text{-}\;Y_{average}\right)}^{2}}\equiv 1\;\text{-}\;\frac{PRESS}{SS}$$ (1)

In Formula (1), Y_obs_ and Y_cal_ represent observed and calculated activity values respectively, while Y_average_ means average activity value of the entire data set. Often, a high q^2^ value (q^2^ > 0.5) is reflected as an evidence of high predictive ability of the QSAR model. The *PRESS* value is referred as ‘Predictive Residual Sum of Squares’, which is the difference between the predicted values Y_cal_ and the observed values Y_obs_. The Sum of Squares (*SS*) denotes to the difference between the observed values Y_obs_ and their mean Y_average_ [48].

$$r^{2}=1\;\text{-}\;\frac{\sum {\left(Y_{cal}^{fit}\;\text{-}\;Y_{obs}\right)}^{2}}{\sum {\left(Y_{obs}\;\text{-}\;Y_{average}\right)}^{2}}\equiv 1\;\text{-}\;\frac{RSS}{SS}$$ (2)

In Formula (2), Y^fit^_cal_ denotes the fitted value calculated with the linear regression. *RSS* refers ‘Residual Sum of Squares’ which is difference between the fitted values Y^fit^_cal_ and the observed values Y_obs_. The numerator term *SS* is the ‘Sum of Squares’ which is difference between the observed values Y_obs_ and their mean values Y_average_. For the reliable QSAR model, r^2^ value should be greater than 0.6 [48], and the difference between r^2^ and q^2^ should not exceed 0.3 [49].

$$r^{2}{}_{pred}=1\;\text{-}\;\frac{\sum {\left[Y_{cal(test)}\;\text{-}\;Y_{obs(test)}\right]}^{2}}{\sum {\left[Y_{obs(test)}\;\text{-}\;Y_{mean\text{-}obs(training)}\right]}^{2}}$$ (3)

In Formula (3), Y_cal(test)_, Y_obs(test)_ and Y_mean-obs(training)_ denote calculated, observed values of the test set and mean values of training set respectively. For a statistically fit QSAR model with good predictive ability, r^2^_pred_ value should be more than 0.5. In Formula (4), Y_obs_, Y_cal_ and n represent observed, calculated activity values and number of compounds respectively [48].

$$S=\sqrt{\frac{\sum {\left(Y_{obs}\;\text{-}\;Y_{cal}\right)}^{2}}{n\;\text{-}\;2}}$$ (4)

$$F_{test}=\frac{\frac{\sum {\left(Y_{cal}\;\text{-}\;Y_{mean}\right)}^{2}}{p}}{\frac{\sum {\left(Y_{obs}\;\text{-}\;Y_{cal}\right)}^{2}}{n\;\text{-}\;p\;\text{-}\;1}}$$ (5)

In Formula (5), Y_obs_, Y_cal_, Y_mean_, n and p denote observed, calculated, mean activity values, number of compounds and predictor variables, respectively. Furthermore, S value closer to 0 and F-test value should be near above threshold value *i*.*e*. larger the F-test value; greater is the probability that QSAR models are statistically significant [48].

## Results and discussion

Computer-aided drug designing tools such as molecular modelling, pharmacophore mapping, molecular docking, homology modelling and QSAR modelling are some of the robust approaches which have been widely employed by the researchers around the world in order to elucidate the target structure or recognize the active sites within the target, construction of the target structure, virtual screening, lead optimization, ligand-target binding affinity prediction, establishment of selectivity of ligands against a particular target, generation of pharmacophore, construction of molecular library bearing active pharmacophore, and more importantly to hasten the drug discovery process. In the present study, integrated molecular docking assisted 3D-QSAR was successfully performed on a data set of 21 compounds comprises of curcuminoids (compound 1–3) and synthetic curcumin analogues (compound 4–21) (Fig 4) to find out binding affinity against ARL2 with the identification of molecular shape and electronic features of molecules responsible for potent ARL2 inhibitory activity. The 3D-QSAR models were built by using self-organizing molecular field analysis (SOMFA) proposed by Robinson and co-workers [5, 40, 50].

**Fig 7:**
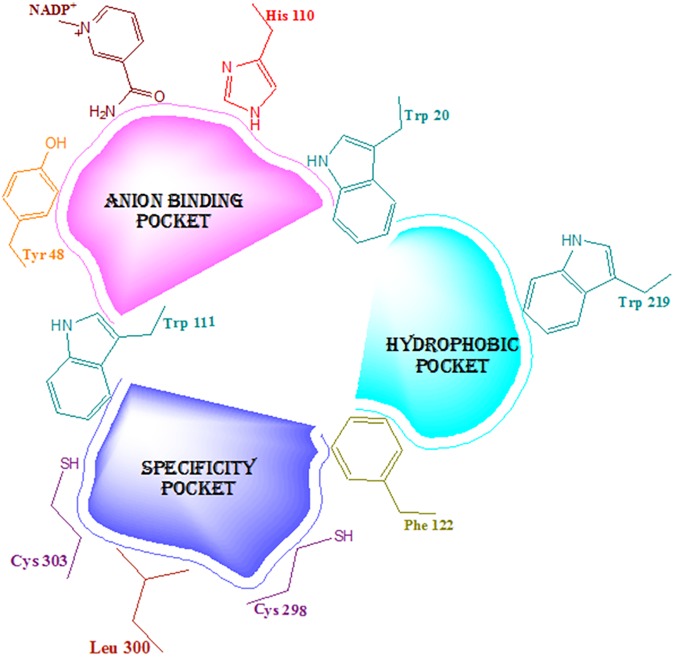
Projected binding pockets in the active site of ALR2 according to previously performed X-ray crystallography and mutagenesis studies [55–59].

Initially, the geometrically optimized data set molecules were subjected to molecular docking simulations with the ALR2 for conformational search as well as detection of mode and extent of binding interactions. ALR2 (EC 1.1.1.21) is a 36 kDa TIM-barrel-shaped aldo-keto reductase consists of a single polypeptide domain of 316 amino acid residues [46, 51]. The polypeptide chain clogged at the amino terminus ends into a β/α-barrel structural motif containing eight parallel β strands which are connected to each other by eight outlying α-helical segments running anti-parallel to the β sheet. The active site is located in a large and deep cleft in the C-terminal end of the β barrel, and the NADPH cofactor binds in an extended conformation to the bottom of the active site [52, 53]. However, it is prospective that the active site often changes its conformational shape depending on the binding conformations of bound ligand. The ligand-dependent conformations of the ALR2 indicate a remarkable induced fit or flexibility of the active site [54]. Three distinct binding pockets (Fig 7) in the active site of ALR2 can be projected according to X-ray crystallography and mutagenesis studies performed on ALR2 [55–59]: (1) ‘anion binding pocket’, made up of Tyr 48, His 110, Trp 20, Trp 111 amino acid residues in union with the positively charged nicotinamide of the cofactor NADP^+^; (2) ‘hydrophobic pocket’ or ‘specificity pocket’ lined by the amino acid residues Leu 300, Cys 298, Cys 303, Trp 111 and Phe 122 [55]; (3) another ‘hydrophobic pocket’ constituted by the amino acid residues Trp20, Trp111, Phe122, and Trp219. The specificity pocket demonstrates a high degree of flexibility and the constructing residues of this pocket are not conserved in other AKRs including ALR1 [57].

Molecular docking (MD) simulations were performed into the cavity 1 having largest surface area (149.76 Å^2^) and volume (51.712 Å^3^) (Table 1) out of a total five cavities (1–5) (Fig 5) searched within the ARL2 (PDB entry: 4JIR) [46]. The key amino acid residues of cavity 1 nearby the proximity of 6.00Å included Ala 299, Arg 296, Cys 298, His 110, Leu 300, Leu 301, Phe 122, Trp 20, Trp 79, Trp 111, Trp 219, Tyr 48, Val 47 and Val 297 (Fig 6).

**Table 1:** Major cavities (1–5) detected in ALR2 along with their volume, surface area, and position.

Cavity No.	Volume (Å^3^)	Surface Area (Å^2^)	Position Co-ordinates (Å)		
			X	Y	Z
1	51.712	149.760	-6.554	7.752	17.722
2	24.064	106.240	-10.182	7.418	24.254
3	22.016	87.040	-8.180	-9.006	26.857
4	15.360	72.960	11.859	-5.531	4.280
5	12.288	48.640	-9.534	14.142	6.100

**Fig 8:**
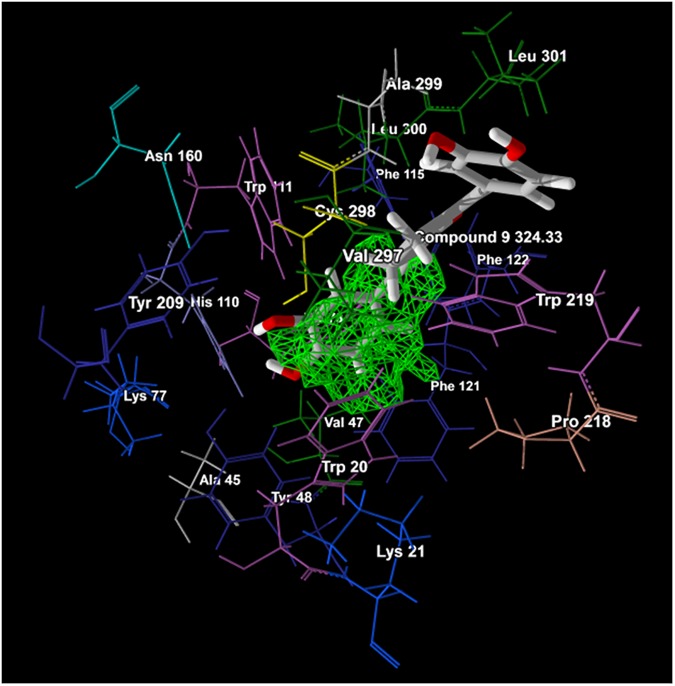
Docking view of compound 9 (Mol. Wt. 324.33) into cavity 1 (green) of ALR2 with its constituting amino acid residues.

MolDock, Re-rank, and H-Bond scores were obtained after the completion of MD simulations (Table 2). The docking view of most active compound 9 with ALR2 is also depicted in Fig 8. The MolDock score shows the quality of binding fitness and plausible orientation of ligand within the active site of the target. The Re-rank score uses a weighted combination of the terms used by the MolDock score mixed with a few addition terms. Re-rank scoring function improves MD simulations accuracy by identifying the most likely docking solution from the solutions obtained by the MD algorithm [5]. The Re-rank score includes the steric (by LJ12-6) terms which are Lennard-Jones approximations to the steric energy while the MolDock score uses a piecewise linear potential to approximate the steric energy. H-bond score demonstrates the strength of H-bond interactions formed between the ligand and ALR2 [5, 40]. The most potent compound 9 (IC_50_ 2.60 μM) among the selected data set showed highest MolDock and Re-rank scores when compared with ARI of natural and synthetic origin namely Quercetin (IC_50_ 37.60 μM) [32] and Epalrestat (IC_50_ 0.17 μM) [60], respectively; designates its greater binding affinity with ALR2. However, H-bond score of Quercetin was higher than the compound 9 (Table 2) due to greater no. of H-bond interactions between Quercetin and ALR2.

**Table 2:** MolDock, Re-rank and H-Bond scores of compound 9, Quercetin and Epalrestat.

Compound ID	MolDock Score (Kcal/Mol)	Re-rank Score (Kcal/Mol)	H-Bond Score (Kcal/Mol)
9	-135.695	-112.341	-6.396
^a^Quercetin	-131.042	-106.527	-2.153
^b^Epalrestat	-123.840	-105.481	-14.389

^a^ARI from natural origin

^b^ARI available in the market.

**Fig 9:**
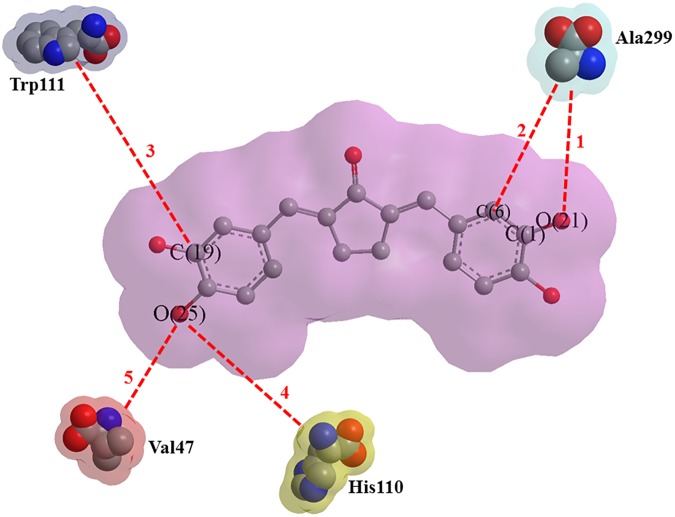
Steric interactions (Id. 1–5, red dashed bonds) of compound 9 with ALR2.

The compound 9 exhibited prominent steric interactions (steric interaction Id. 1, 2, 3, 4, and 5) (Fig 9) with ALR2. The C-6, C-19, O-21 and O-25 atoms of compound 9 are the contributors towards steric interactions with different amino acid residues of ALR2. The amino acid residue Ala 299 share two, His 110, Trp 111 and Val 47 share one steric interaction each with compound 9. Further, His 110 showed most prominent steric interaction (strength 3.31, bond length 2.75 Å) with O-25, and Trp 111 demonstrated distant as well as weaker steric interaction (strength 0.70, bond length 3.19 Å) with C-19 of compound 9 (Table 3).

**Table 3:** Description of steric interactions shown by compound 9 with ALR2.

Steric Interaction ID	Strength	Length (Å)	Contribution in Steric Interaction	
			Ligand	Target
1	0.81	3.17	O-21	Ala 299
2	1.91	2.98	C-6	Ala 299
3	0.70	3.19	C-19	Trp 111
4	3.31	2.75	O-25	His 110
5	2.78	2.48	O-25	Val 47

**Fig 10:**
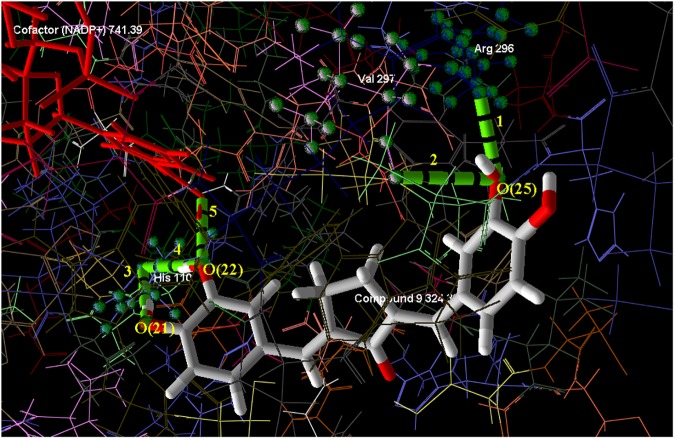
Hydrogen bond interactions (H-bond Id. 1–5, green dashed bonds) of compound 9 with ALR2.

The compound 9 also demonstrated five intermolecular hydrogen bond (H-bond) interactions within the cavity 1 of ALR2 (H-bond Id. 1–5), of which four (H-bond Id. 1, 3, 4 and 5) are prominent with greater energy (-2.5 Kcal/mole Å) and one (H-bond Id. 2) is weaker with lesser energy (-1.5 Kcal/mole Å). The amino acid residue His 110 connects with ligand 9 *via* two simultaneous intermolecular H-bonds formed with atoms O-21 and O-22 (H-bond Id. 3, 4) (Fig 10). The bond length, bond energy and H-Bond donor property of H-Bonds formed between compound 9 and ALR2 are presented in Table 4. Further, the atoms O-22, O-25 of compound 9 exhibited distinct, strong and equal strength H-bonds (bond energies = -2.50 Kcal/mole Å) with cofactor NADP^+^ and Arg 296 (Bond Id. 5 and 1, respectively). The weakest H-bond interaction ((Bond Id. 2, bond energy = -1.504 Kcal/mole Å) exhibited between O-25 of ligand 9 and Val 297 of target ALR2.

**Table 4:** Description of H-bond interactions shown by compound 9 with ALR2.

H-Bond ID	H-Bond Donor	Energy (Kcal/mole Å)	Length (Å)	Contribution in H-Bond	
				Ligand	Target
1	Ligand	-2.500	2.693	O-25	Arg 296
2	Ligand	-1.504	3.212	O-25	Val 297
3	Ligand	-2.500	2.989	O-21	His 110
4	Ligand	-2.500	3.080	O-22	His 110
5	Ligand/Target	-2.500	2.728	O-22	NADP^+^

Minimum binding energy conformations of the selected data set molecules obtained from MD simulations were used for the present 3D-QSAR analysis. The data set molecules were aligned using two different alignment approaches based on molecular weighted (MW) extent and moments of inertia (MI). The grid spacing of 1.0 Å and 0.5 Å both were investigated in the present study for the generation of 3D-QSAR models on MW and MI aligned molecules. The shape and electrostatic master grid maps generated from 3D-QSAR were used to predict the molecular shape and electronic contribution of individual molecules towards ALR2 inhibitory activity. The shape and Electrostatic potential were combined using PLS regression to develop a statistically reliable 3D-QSAR model. Statistical summary of the developed 3D-QSAR models (1–4) for the series of curcuminoids and curcumin analogues is presented in Table 5, among them Model 2 based on MW alignment pattern with 1.0 Å grid resolutions showed best statistical quality and is considered for the present 3D-QSAR study. The QSAR models (1) following MW alignment approaches at 0.5 Å grid resolutions also produce a good correlation with slightly inferior statistical fitness as compared to model 2 developed at 1.0 Å grid resolutions (Table 5). The 3D-QSAR model 2 showed good non-cross validated correlation coefficient r^2^ (0.609), cross-validated correlation coefficient q^2^ (0.564), S-value (0.250), predictive correlation coefficient r^2^_Pred_ (0.736), F-test value above a threshold value *i*.*e*. 24.945, hence used for predicting ALR2 inhibitory activities of the curcuminoids and synthetic curcumin analogues. The contribution of electrostatic and shape potential are 50.5% and 49.5%, respectively, indicates that the electrostatic and shape potential of data set molecules are of nearly equal importance for ALR2 inhibitory activity. Observed, predicted and residual activities of all the molecules are reported in Fig 4 using best 3D-QSAR Model 2. S1 Fig depicts a good linear correlation and the moderate difference between actual and predicted values of molecules in the training and test set of selected data set.

**Table 5:** Statistical quality of 3D-QSAR models generated at 0.5 Å and 1.0 Å grid resolutions using PLS regression with different alignment approaches.

3D-QSAR Model	Alignment	Descriptor	Grid Resolution	r^2^	q^2^	S	r^2^_Pred_	F-test	QSAR Equation
1	MW	Shape + Electrostatic	0.5 Å	0.597	0.550	0.254	0.765	23.694	pIC_50_ = 0.5021 Shape + 0.5023 Electrostatic + 0.0054
**2**	MW	Shape + Electrostatic	1.0 Å	**0.609**	**0.564**	**0.250**	**0.736**	**24.945**	pIC_50_ = 0.5024 Shape + 0.5030 Electrostatic + 0.0066
3	MI	Shape + Electrostatic	0.5 Å	0.581	0.535	0.259	0.440	22.197	pIC_50_ = 0.4988 Shape + 0.5055 Electrostatic + 0.0050
4	MI	Shape + Electrostatic	1.0 Å	0.592	0.546	0.256	0.486	23.167	pIC_50_ = 0.4992 Shape + 0.5054 Electrostatic + 0.0056

**Fig 11:**
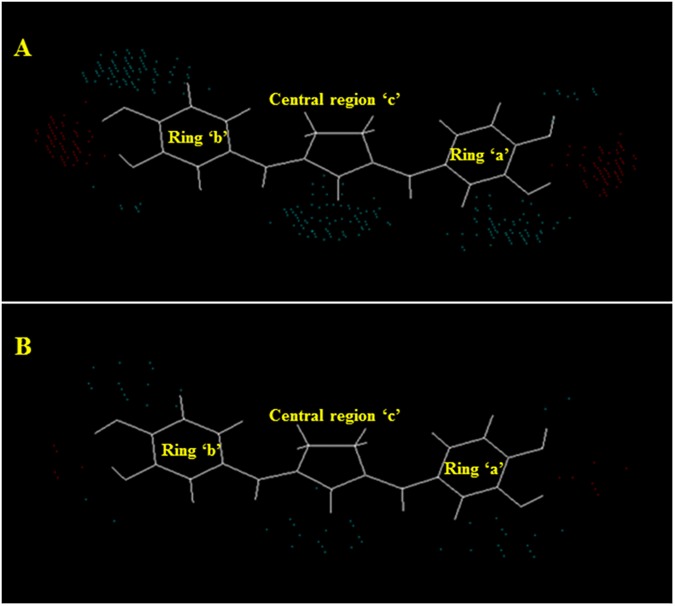
Electrostatic master grid maps at 0.5 Å (A) and 1.0 Å (B) grid resolutions.

**Fig 12:**
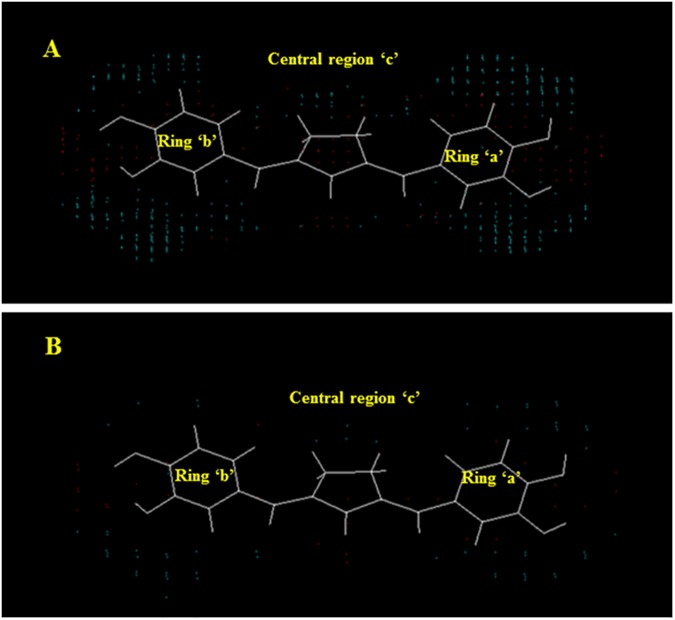
Steric master grid maps at 0.5 Å (A) and 1.0 Å (B) grid resolutions.

For the better insight into essential 3D-pharmacophoric features (Electronic and shape) of curcumin analogues for potent and selective ALR2 inhibitory activity, respective electrostatic and steric master grid maps were generated from 3D-QSAR models (1 and 2) at both the grid resolutions 0.5 Å and 1.0 Å (Figs 11 and 12). Master grid maps are the 3D maps that represent the relationships between molecular properties and biological activity. These maps are the clear indicator for predicting and designing novel molecules with improved potency profile against a putative target along with maintaining selectivity. Critical interpretation of master grid maps led to the identification of key structural features that could be exploited for improving the potency of the most potent reference compound. Furthermore, the master grid maps provide a direct graphic indication regarding structural topographies accountable to differentiate the activities of molecules present in the training set under study [5]. In the present study, most potent synthetic curcumin analogue (compound 9) was used as a reference structure for visualization of master grid maps. For the interpretation of electrostatic and steric master grid maps, the compound 9 was divided into three distinct regions: (1) central region ‘c’, (2) ring ‘a’, and (3) ring ‘b’ (Figs 11 and 12).

In electrostatic master grid map (Fig 11), red points denote the electrostatic favoured area indicating more positive charge increases activity, and blue points represent un-favoured area indicating more negative charge increases activity [40]. The presence of high density of blue points around oxygen atom of >C = O group of the centrally positioned cyclopentanone ring (c) indicates that curcumin analogue should hold doubly bonded electronegative negative atom or group at the centre of the molecule for favourable activity. Presence of blue points at the oxygen atoms and cluster of red points around hydrogen atoms of the -OH groups at ‘R_1_’ of ring ‘a’, both the ‘R_1_’ and ‘R_2_’ of ring ‘b’ signifies the importance of bivalent electronegative atom/group directly attached with rings ‘a’ and ‘b’ at the respective positions, remaining valency of the bivalent electronegative atom/group should be satisfied with electropositive atoms/groups for potent ALR2 inhibitory activity. Furthermore, few blue points around the substituent ‘R_2_’ of ring ‘a’ indicate slight importance of electronegative group at this position for favourable activity.

In steric master grid map (Fig 12), red points denote the sterically favoured area indicating more steric bulk increases activity, and blue points represent un-favoured area indicating more steric bulk decreases activity [40]. The Presence of a high density of red points above the plane of ring ‘c’ and below the plane of ring ‘a’, ‘b’ indicates bulky core skeleton is favourable. The occurrence of few red points in the vicinity of the cluster of blue points around the substituents ‘R_1_’, ‘R_2_’ of both the rings ‘a’ and ‘b’ describe the importance of non-bulky atoms/groups with very short branching are favorable at these positions. A cluster of blue points around substituents ‘R_3_’ of both the ring ‘a’ and ‘b’ suggesting sterically non-bulky atom/group is strongly favorable. The few blue points around the two substituents remaining except ‘R_1_’, ‘R_2_’, ‘R_3_’ at both the rings ‘a’ and ‘b’ implies that these positions should be non-branched or substituted with non-bulky atoms/groups for favourable activity. The central ring ‘c’ is peripherally surrounded by blue points suggests that central region without branching is favourable for ALR2 inhibitory activity. Furthermore, few blue points in conjugation of red points around the oxygen atom of >C = O group of the centrally positioned cyclopentanone ring (c) indicate that the bivalent atom may be with very short branching like in curcuminoids or without branching for favourable activity.

In addition, all the data set molecules (1–21) were also screened for drug-likeness or ADME screening based on ‘Lipinski’s Rule of Five’, which state that for becoming an orally active drug candidate a molecule should have no more than one violation of the following criteria: molecular weight ≤ 500, Log P ≤ 5, H-Bond donor ≤ 5, H-bond acceptor ≤ 10, and rotatable bonds ≤ 10 [44]. The ADME screening data shows that all the data compounds obey drug- likeness parameters except compound 8 (molecular wt. = 524.22, Log P = 5.041) (Fig 4).

## Conclusion

**Fig 13:**
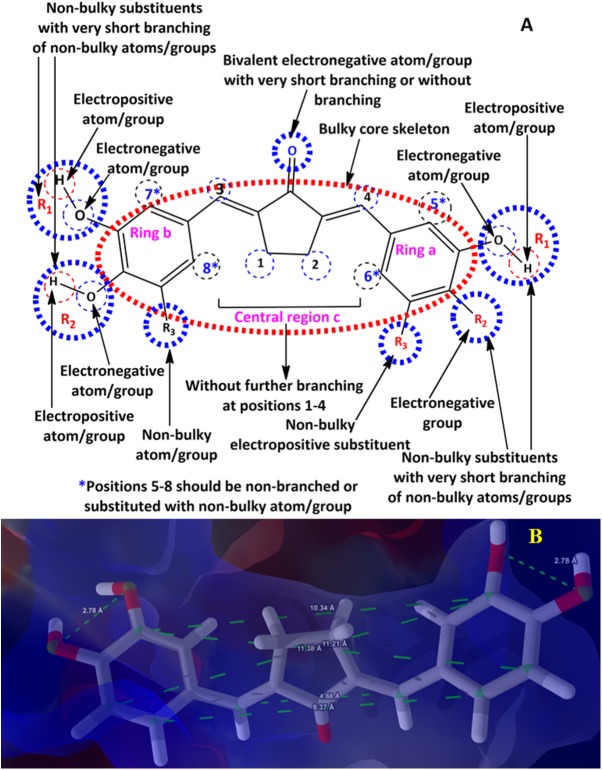
Pharmacophoric frameworks or spatial fingerprints (A and B) of curcumin analogues having favourable interactions with ALR2.

In current years, rigorous work has been done in search of appropriate therapeutic agents of natural origin and their synthetic analogues active against ALR2 for the management of diabetic complications. However, except Epalrestat, none of the synthetic or natural analogue is presently available in the market due to lack of target selectivity, poor pharmacokinetic and potency profile. At present, 3D-QSAR techniques, for example, CoMFA, CoMSIA and SOMFA are widely used for the structural, functional and steric modifications of a chemical scaffold so as to develop new molecules with greater selectivity and improved pharmacokinetic and potency profile against a biological target. In addition to 3D-QSAR techniques, MD simulations are also commonly used by researchers around the world to expose the lowest energy conformations of molecules under investigation, potentially explore their binding interactions and predict binding affinity with the key amino acid residues present in the active site or binding cavity of the target. In our present work, molecular docking assisted 3D-QSAR models on a data set of curcuminoids and their synthetic analogues active against ALR2 have been developed using method based on two different alignment approaches namely molecular weighted (MW) extent and moments of inertia (MI) in order to map the spatial fingerprints/pharmacophoric features of curcumin analogues for their ALR2 selectivity, potency, and better pharmacokinetic profile. Among all the generated 3D-QSAR models (1–4, Table 5), model 2 possessed good internal and external consistency and showed statistical significance and predictive abilities. Based on the cross-validation results, it can be perceived that model 2 based on MW alignment with 1.0 Å grid resolution has almost similar predictive capability with model 1 at 0.5 Å grid resolution, and better predictability than both models (3 and 4) based on MI alignment. Moreover, the model 2 with r^2^ = 0.609, Q^2^ = 0.564, S = 0.250, r^2^_Pred_ = 0.736 and F-test = 24.945, indicating it is reliable enough for activity prediction and explained the potent ALR2 inhibitory activity of compound 9. The master grid maps obtained from 3D-QSAR indicated significant electrostatic and shape potential contributions and suggested sufficient information for understanding the structure-activity relationship, thus aided in the further design and development of novel curcumin analogues with ALR2 selectivity and improved potency profile. The amalgamated pharmacophoric frameworks or spatial fingerprints of curcumin analogues necessary for selective and potential ALR2 inhibitory activity are presented in Fig 13 and can be mapped for future pharmacophoric modifications of curcumin analogues for the development of novel, more potent and ALR2 selective molecules with the steady pharmacokinetic profile. Furthermore, the drug-likeness or ADME screening results strongly support that curcumin analogues can be proposed as a good drug candidate in this domain.

## Supporting information

S1 Fig Plot of actual vs. predicted activities for training and test set molecules obtained from the best 3D-QSAR model 2. (TIF)
