# Supplementary figures and images for: Structure based comprehensive modelling, spatial fingerprints mapping and ADME screening of curcumin analogues as novel ALR2 inhibitors
Source: PLoS One. 2017 Apr 11;12(4):e0175318. doi: 10.1371/journal.pone.0175318 (PMC5388491; doi:10.1371/journal.pone.0175318)

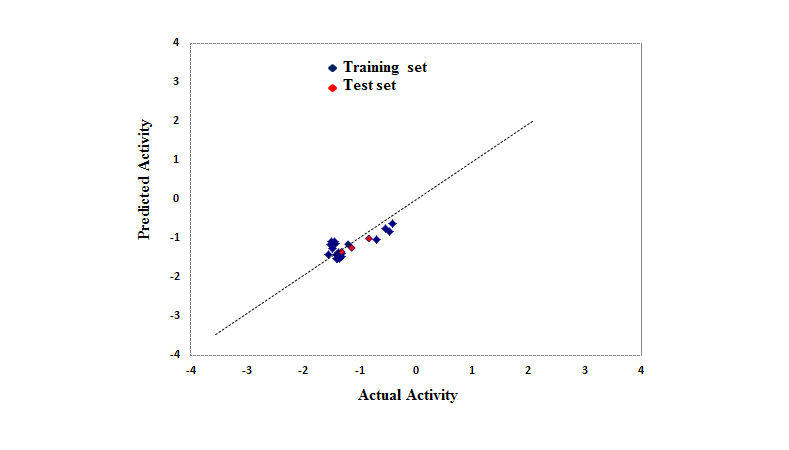

Supplement: S1 Fig — (TIF) [file pone.0175318.s001.tif]
